# Quercetin Released Biomedical Hybrid Hydrogels Fabricated by Silk Fibroin and Sodium Alginate with Incorporation of Ag@rGO Nanosheets

**DOI:** 10.3390/molecules31030527

**Published:** 2026-02-03

**Authors:** Lei Nie, Xinran Li, Benda Xing, Ling Wang

**Affiliations:** College of Life Sciences, Xinyang Normal University, Xinyang 464000, Chinawangling@xynu.edu.cn (L.W.)

**Keywords:** silk fibroin, alginate, quercetin, Ag@rGO nanosheets, hydrogels

## Abstract

The drug-encapsulated hybrid hydrogels possessed several expected properties, including porous microstructure, conductivity, adhesive strength, antioxidant activity, antibacterial properties, and cytocompatibility, and have great potential in biomedical applications, such as skin wound hydrogel dressings and bio-adhesives. In this paper, the quercetin-loaded hybrid hydrogels (SSA-QRs) were fabricated using silk fibroin (SF), alginate, and silver-doped reduced graphene oxide (Ag@rGO) nanosheets, incorporating quercetin-encapsulated PF-127 (PF127-QR) micelles. Scanning electron microscopy (SEM) images confirmed that the fabricated hybrid hydrogels possessed an interconnected porous microstructure. The mechanical properties of hydrogels could be regulated by adjusting the content of incorporated Ag@rGO nanosheets and PF127-QR micelles. Furthermore, the obtained SSA-QR hydrogels displayed the expected swelling properties, and the swelling rates could reach 1200–1700% in 120 min, in the equilibrium state. The fabricated SSA-QR hydrogels possessed apparent conductivity and self-healing ability. In addition, SSA-QR hydrogels exhibited strong adhesive performance on the surface of different materials, including skin, metal, wood, plastic, and glass. The typical antibacterial testing using Gram-positive *Staphylococcus aureus* (*S. aureus*) and Gram-negative *Escherichia coli* (*E. coli*) confirmed the excellent antibacterial activities of SSA-QR hydrogels. Moreover, SSA-QR hydrogels displayed good antioxidant ability and intracellular ROS scavenging ability. However, the increased content of Ag@rGO nanosheets could cause a great increase in the hemolysis ratio for SSA-QR hydrogels. Fluorescent images, cell counting kit-8 (CCK-8) assay, and cell scratch testing confirmed their excellent cytocompatibility and cell pro-migration ability. The available results demonstrated a facile strategy to prepare the quercetin-loaded hydrogel for applications of wound hydrogel dressing and bio-adhesives.

## 1. Introduction

As soft viscoelastic materials, hydrogels composed of three-dimensional (3D) polymeric networks have been garnered in many biomedical applications, including drug delivery systems, tissue regeneration, biosensors, and wound dressing [1]. Specifically, the ability to mimic the extracellular matrix (ECM) offers hydrogels a suitable biochemical environment to support the cellular interactions [2]. In recent years, more functional hydrogels with additional features have been introduced. These features mainly include drug release control, conductivity, adhesive ability, antibacterial properties, antioxidant activity, reactive oxygen species (ROS) scavenging, and cell pro-migration ability [3]. The adaptability of hydrogels makes them continue at the forefront of materials innovation in biomedicine and tissue engineering applications. Many original natural polymers can be used to fabricate hydrogels; these polymers are mainly derived from polysaccharides and proteins [4,5]. In particular, polysaccharides can be used to design engineered hydrogels via physical noncovalent or chemical covalent cross-linking approaches, owing to their intrinsic functional moieties [6]. Polysaccharides mainly include chitosan, cellulose, alginate, hyaluronic acid (HA), xanthan, etc. [7]. Protein polymers include collagen, elastin, silk fibroin (SF), albumin, etc. [3,8].

As a burgeoning silk fibroin-based hydrogel, the unique biocompatibility and mechanical strength make it a marvel in biomedical fields [9,10]. Silk is produced by silkworms or spiders. Silk has a complex structure consisting of a heavy (H) chain and a light (L) chain linked by disulfide bonds [11]. SF can be obtained from silk via degumming and purification. SF is also considered a safe, more acceptable biomaterial, with no concerns about religious restrictions or disease transmission risks [12]. As the main structure in SF, several amino acids are present in the primary structure of the heavy chain, such as glycine, alanine, serine, and tyrosine. In addition, the stacked β-crystallites were interspersed in an amorphous matrix and the non-repetitive domains are formed [13]. SF-based hydrogels are generally fabricated by using SF in an amorphous aqueous solution as a precursor, allowing the second polymer network to be easily introduced to form composite hydrogels.

Among polysaccharides, alginate, as a natural anionic hydrophilic polymer, has been employed in a wide range of biomedical applications [14]. Alginate, obtained from bacteria and brown algae, is a linear polymer that consists of α-mannuronic acid and β-guluronic acid connected via 1,4 glycosidic bonds [15]. Due to the inherent properties of promoting cell growth and differentiation, alginate-based materials (hydrogels) have been used in tissue engineering and artificial organ applications [16]. Combining alginate and SF to fabricate a biomedical hydrogel could avoid using chemically derived cross-linking agents, such as carbodiimide coupling and glutaraldehyde. The obtained hybrid materials could also benefit from the synergistic effects of SF and alginate, with ECM biomimicry improved and mechanical strength regulated by varying SF/alginate ratios [17]. Due to the rapid gelation by calcium alginate formation, SF/alginate composite hydrogel could be used to encapsulate cells as bioinks for bioprinting, such as inkjet bioprinting [18]. The SF/alginate hydrogel containing 2 *w*/*v*% SF and 1 *w*/*v*% sodium alginate could form uniform strands and support high cell viability [19]. It is interesting that incorporating graphene oxide (GO) or casein hydrolysate into an SF/alginate hybrid hydrogel can further greatly improve its biological properties and mechanical strength. The introduction of GO could especially make the hydrogel have good conductivity, and the cell behavior, such as cell pro-migration ability, can be modulated [20,21]. S. Yang et al. have reported a material-centric SF/alginate/GO sponge with an ECM-like structure for acellular and bone regeneration [22]. Considering that GO could be covalently connected to sodium alginate polymer, the composite SF/alginate/GO hydrogels have exhibited enhanced mechanical properties. However, the poor antibacterial abilities of composite hydrogels still limit their biomedical applications, such as bio-adhesives and wound healing dressing.

During tissue regeneration, reactive oxidants or free radicals produced under oxidative stress can damage biomolecules in cells, such as lipids, proteins, and deoxyribonucleic acid (DNA) [23]. Biomedical hydrogels that can scavenge excess ROS or reactive nitrogen species (RNS) for tissue engineering applications could accelerate tissue regeneration [24]. Integrating growth factors and antioxidants into biomedical hydrogels is an effective approach to fabricating antioxidant hydrogels. Quercetin, 3,3′,4′,5,7-pentahydroxyflavone, a natural active flavonoid, is derived from many plants, such as onions, citrus fruits, tea, peppers, broccoli, and camellias [25]. Quercetin possesses a range of expected biological activities, including antioxidant, antibacterial, antifungal, anti-inflammatory, antiviral, and antitumor activities [26]. However, the poor water solubility and low stability of quercetin limit its loading and controlled release in hydrogels. Encapsulation of quercetin in polyether F127 (Pluronic F127) micelles has been shown to be a practical approach for loading and releasing quercetin [27,28].

**Scheme 1:**
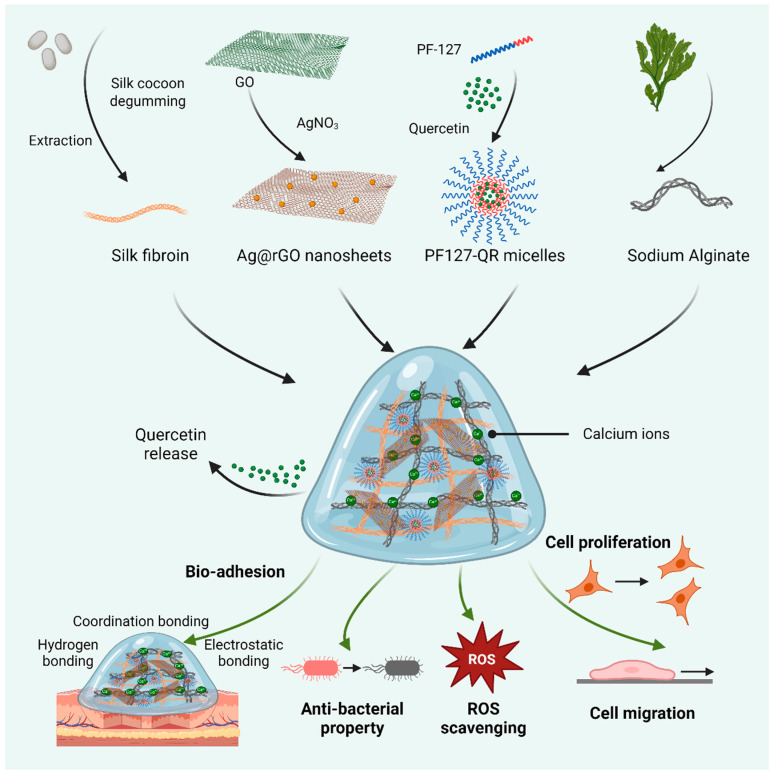
Schematic diagram of preparing biomedical hydrogels based on the synthesized silk fibroin (SF) and alginate, reinforced by silver-doped rGO (Ag@rGO) nanosheets. In addition, quercetin was loaded in PF-127 micelles for incorporation into hydrogels. The related properties of quercetin-released hydrogels, including adhesive ability, ROS scavenging, antibacterial activity, and cytocompatibility, were investigated.

In this work, we have employed an approach of doping silver nanoparticles onto reduced graphene oxide (rGO) nanosheets to improve their antibacterial activity [29]. Next, the biomedical hybrid hydrogels were designed based on silk fibroin, alginate, and the silver-doped rGO (Ag@rGO) nanosheets. At the same time, quercetin was loaded in PF-127 micelles to obtain PF127-QR micelles. Then, quercetin-encapsulated composite hydrogels were fabricated using SF, alginate, Ag@rGO nanosheets, and PF127-QR micelles, as shown in Scheme 1. By combining the intrinsic and synergistic effects of Ag@rGO nanosheets and quercetin, the composite hydrogels were expected to exhibit enhanced antioxidant, ROS scavenging, and antibacterial activities. In addition, the physicochemical and biological properties of composite hydrogels were investigated, including microstructure, dynamic swelling rate, quercetin release profile, conductivity, self-healing behavior, adhesive ability, cytocompatibility, and cell pro-migration ability.

## 2. Results and Discussion

### 2.1. Synthesis of PF127-QR Micelles

**Figure 1:**
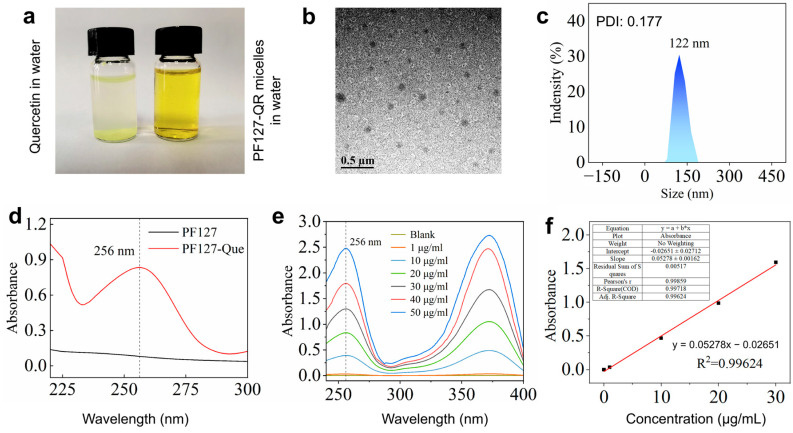
(**a**) The prepared PF127-QR micelles were dissolved in water. (**b**) TEM image of PF127-QR micelles. (**c**) DLS analysis of PF127-QR micelles; the sample was dispersed in water. (**d**) UV–vis absorbance spectra of PF127 micelles and PF127-QR micelles. (**e**) UV–vis absorbance curves of curcumin using different concentrations dissolved in methanol solutions. (**f**) The simulated standard absorption curve of quercetin.

The hydrophobic drug, quercetin, can be loaded in Pluronic P127 copolymer micelles [30]. It is clearly observed that quercetin was not dissolved in water, and quercetin-loaded PF127 micelles were completely dispersed in water (Figure 1a). The morphology of the synthesized PF127-QR micelles could be observed using TEM image (Figure 1b). The spherical micelles were formed. According to the dynamic light scattering (DLS), the hydrodynamic diameter of PF127-QR micelles was around 122 nm (PDI = 0.177) (Figure 1c), indicating a narrow size distribution and excellent colloidal stability. The successful encapsulation of quercetin in PF-127 micelles could be confirmed using a UV–vis spectrophotometer. The typical peak of 256 nm for quercetin was detected in the UV–vis curve of PF-127 micelles (Figure 1d). Next, the encapsulation efficiency (*EE*) and drug loading (*LC*) of quercetin in PF127 micelles were tested. According to the UV–vis absorbance values of quercetin using different concentrations, the standard absorption curve of quercetin was obtained (Figure 1e,f). In this work, the calculated EE of quercetin was 75.30 ± 1.20%, and the LC of quercetin was 8.60 ± 0.58%.

### 2.2. Fabrication of Ag@rGO Nanosheets

**Figure 2:**
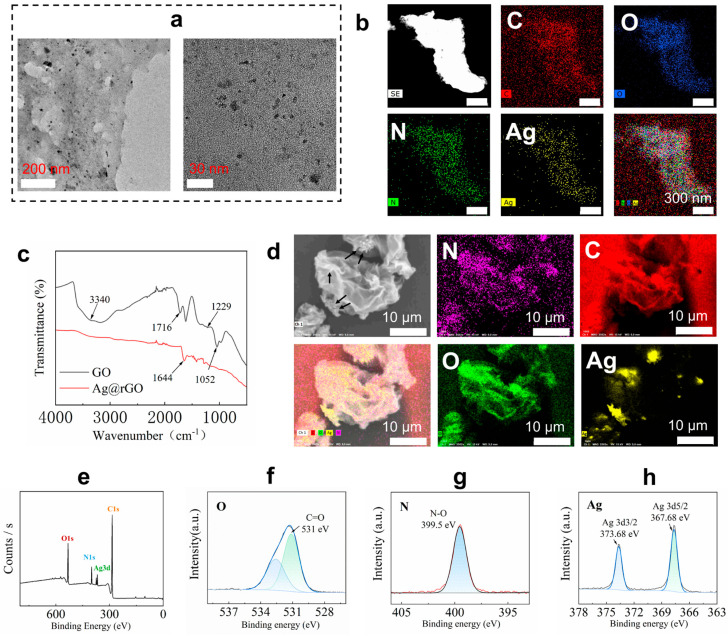
(**a**) TEM images of Ag@rGO nanosheets at different magnifications. (**b**) Element mapping images of Ag@rGO nanosheets. (**c**) FT-IR spectra of the prepared Ag@rGO and GO nanosheets. (**d**) SEM image of Ag@rGO nanosheets and element mapping images analysis; arrows indicate the formed silver nanoparticles. (**e**) XPS spectrum of Ag@rGO nanosheets. (**f**) O1s scanning. (**g**) N1s scanning. (**h**) Ag3d scanning of Ag@rGO nanosheets.

The silver nanoparticles were formed on the surface of rGO nanosheets. The synthesized Ag@rGO nanosheets were characterized using TEM images, FT-IR, SEM images, and XPS, as shown in Figure 2. From the TEM images of Ag@rGO nanosheets, the silver nanoparticles were anchored on the formed rGO nanosheets. No agglomeration was observed (Figure 2a). The size of silver nanoparticles measured from TEM images was around 5–8 nm. It was interesting that less stacking of rGO sheets occurred because the anchored silver nanoparticles enhanced the surface area of the rGO nanosheets [31]. Elemental mapping analysis indicated that elements C, O, N, and Ag were present in Ag@rGO nanosheets without detecting impurities (Figure 2b). From the FT-IR spectra of GO and Ag@rGO nanosheets, the peaks at 1716 cm^−1^ belonging to GO disappeared in the spectrum of Ag@rGO nanosheets, due to the reduction in GO (Figure 2c). In addition, the morphology of Ag@rGO nanosheets was also observed using SEM image (Figure 2d), the deposited silver nanoparticles were displayed, and the element mapping analysis confirmed the successful formation of silver nanoparticles on rGO nanosheets. Furthermore, XPS was employed to analyze Ag@rGO nanosheets (Figure 2e–h). In the XPS spectrum of Ag@rGO nanosheets (Figure 2e), typical peaks, O1s, C1s, N1s, and Ag3d, were observed. Due to the formation of Ag0, two peaks at 373.68 (Ag3d3/2) and 367.68 eV (Ag3d5/2) were clearly detected (Figure 2h). The above results confirmed the successful synthesis of Ag@rGO nanosheets.

### 2.3. Physicochemical Properties of Hydrogels

**Figure 3:**
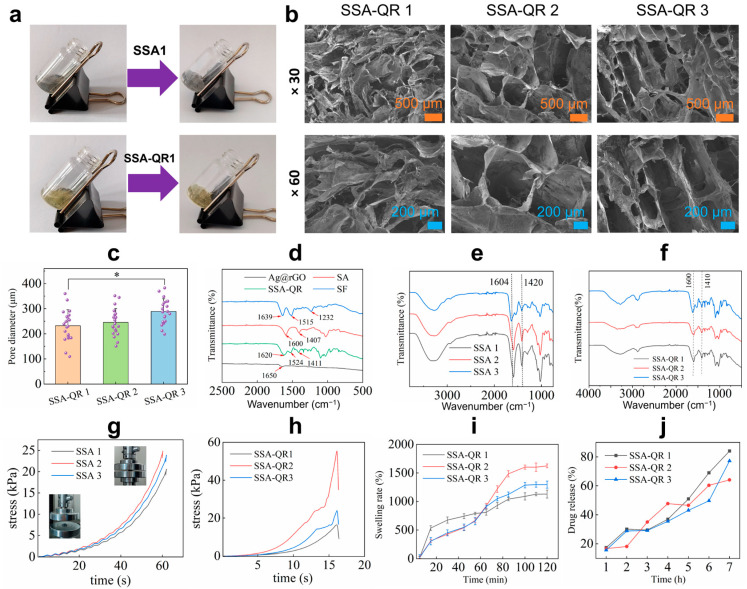
(**a**) Photos display the sol–gel transfer of SSA and SSA-QR hydrogels. (**b**) SEM images of SSA-QR hydrogels, the magnification for the upper SEM images was ×30, and the magnification for the lower SEM images was ×60. (**c**) Pore diameter distribution of SSA-QR hydrogels statistically analyzed from five SEM images per sample using ImageJ software (Version 1.53), * *p* < 0.05. (**d**) FT-IR spectra of Ag@rGO nanosheets, silk fibroin, alginate, and SSA-QR hydrogel. FT-IR spectra of (**e**) SSA hydrogels and (**f**) SSA-QR hydrogels. The stress–strain curves of (**g**) SSA hydrogels and (**h**) SSA-QR hydrogels in compressive strength testing, and the photos inserted in (**g**) indicate the sample (SSA-1) before and after testing. (**i**) The dynamic swelling rates of the SSA-QR hydrogels immersed in PBS. (**j**) The release profiles of quercetin from SSA-QR hydrogels in PBS.

Based on silk fibroin, alginate, and Ag@rGO nanosheets, SSA hydrogels were fabricated. With the incorporation of quercetin-loaded PF-127 micelles, quercetin-loaded SSA (SSA-QR) hydrogels were obtained. SSA and SSA-QR hydrogels were quickly formed from pre-solutions; it took around 5–10 s for the sol–gel transition for both kinds of hydrogels, as shown in Figure 3a. The porous microstructure of hydrogels was investigated using SEM images (Figure 3b). SSA-QR hydrogels exhibited interconnected pores, and SSA hydrogels displayed a similar microstructure to SSA-QR hydrogels. Upon incorporation of PF127-QR micelles into SSA hydrogels, the microstructure remained unchanged. Based on SEM images, the pore diameter distribution of hydrogels could be statistically analyzed using ImageJ (Version 1.53). Five SEM images were randomly selected for each sample. The pores with clear boundaries were manually traced and measured using ImageJ. As shown in Figure 3c, with increasing the content of silk fibroin and Ag@rGO nanosheets, the pore size of hydrogels increased gradually. The chemical interactions between polymers, nanosheets, and micelles were investigated using FT-IR analysis. From the FT-IR spectrum of silk fibroin (Figure 3d), the peaks belonging to the peptide backbones of amide I and amide II (1700–1500 cm^−1^) were detected, indicating the secondary structure of silk fibroin. A couple of peaks appeared, such as 1639 cm^−1^ (β-sheet) for amide I, 1515 cm^−1^ (random coil) for amide II, and 1232 cm^−1^ (random coil) for amide III, indicating the conformations of silk fibroin [32,33]. In the FT-IR spectrum of alginate (Figure 3d), the peaks attained at 1090–1030 cm^−1^ were attributed to C–O stretching of the pyranosyl ring and C–O–C asymmetric stretching, typical structure of alginate saccharide [34]. Strong peaks at 1600 cm^−1^ and 1407 cm^−1^ are designated symmetric and asymmetric stretching vibrations of carboxylate anions [35]. As a result, calcium ions form a chelating structure and the sodium ions are replaced by calcium ions, leading to a shift in the FT-IR spectra of SSA (Figure 3e) [36]. The previous results indicated that quercetin was physically encapsulated in PF-127 micelles and no chemical reactions occurred between quercetin and PF-127 polymer [30,37]. With the incorporation of PF127-QR micelles in SSA hydrogels, several peaks in the range of 1600–1100 cm^−1^ appeared, assigned to the cyclobenzene skeleton structure from quercetin (Figure 3f). According to the FT-IR analysis, PF127-QR micelles were physically present in SSA hydrogel.

The mechanical properties of SSA and SSA-QR hydrogels were investigated. The compressive stress–strain curves of SSA and SSA-QR hydrogels were recorded during the compressive testing, as shown in Figure 3g,h. Sample SSA-1 hydrogel with the lowest content of silk fibroin and Ag@rGO nanosheets exhibited the lowest stress compared to SSA-2 and SSA-3 hydrogels. With increasing silk fibroin and Ag@rGO nanosheet content, the compressive stress of the SSA hydrogel first increased and then decreased. This is likely attributable to the increased viscosity at higher nanofiller loadings, which impedes uniform dispersion. Moreover, excessive silk fibroin and Ag@rGO may competitively interfere with the cross-linking between silk fibroin and sodium alginate, ultimately weakening the hydrogel network, indicating that the highest compressive stress of the hydrogel needs a balance of silk fibroin and Ag@rGO nanosheets. When incorporating PF127-QR micelles, SSA-QR hydrogels exhibited a similar mechanical behavior to SSA hydrogels. Sample SSA-QR2 showed the highest compressive strength compared with SSA-QR1 and SSA-QR3 hydrogels (Figure 3h). In addition, the dynamic swelling behavior of SSA-QR hydrogels was investigated (Figure 3i). Sample SSA-QR1 hydrogel exhibited the fastest swelling rate during the first 60 min compared to other samples. However, the SSA-QR2 hydrogel exhibited the highest swelling rate at equilibrium. According to the quercetin release testing, quercetin displayed similar release profiles from all SSA-QR hydrogels (Figure 3j). The release of quercetin from all SSA-QR hydrogels could reach over 95% after 3 days. Upon reaching the plateau of swelling, drug release does not cease; instead, it proceeds in a sustained and steady manner. This observation unequivocally demonstrates that after the gel network attains a dynamic swelling equilibrium, the micelles—functioning as discrete and governable release units—assume predominant control over the overall drug delivery kinetics through their intrinsic release mechanism.

### 2.4. Conductivity and Self-Healing Behavior

**Figure 4:**
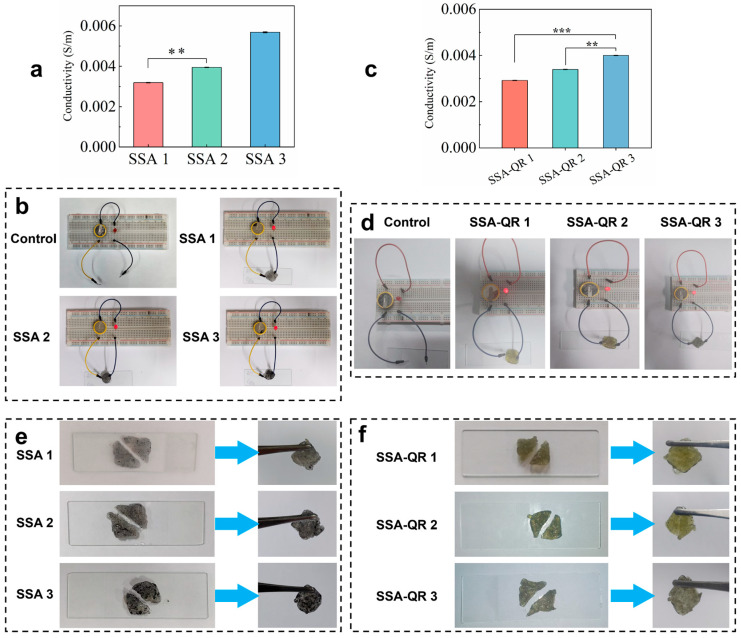
The conductivities of (**a**,**c**) SSA hydrogels and (**b**,**d**) SSA-QR hydrogels, ** *p* < 0.01 and *** *p* < 0.001. In addition, the conductivity of hydrogels was observed using the self-made circuit; the light of the bulb indicates the conductivity. The self-healing behavior of (**e**) SSA hydrogels and (**f**) SSA-QR hydrogels was investigated by reconnecting the cut hydrogels.

The conductivity of hydrogels plays an important role in tissue repair, such as skin, nerve, and bone tissue regeneration [38]. The conductivity of hydrogels was measured using an RTS-8 four-probe tester. With increasing Ag@rGO nanosheets content in hydrogels, the conductivity of SSA hydrogels significantly increased from (3.19 ± 0.11) × 10^−3^ S/m (SSA 1) to (5.69 ± 0.2) × 10^−3^ S/m (SSA 3), while the conductivity of SSA-QR hydrogels also increased from (2.97 ± 0.22) × 10^−3^ S/m (SSA-QR 1) to (3.89 ± 0.16) × 10^−3^ S/m (SSA-QR 3) (Figure 4a,c). The conductivity of the hydrogels was further evaluated using a self-made circuit consisting of a light bulb and a breadboard. Under a 1.5 V power supply, all SSA and SSA-QR hydrogels could illuminate the light bulb when connected to the circuit (Figure 4b,d). With the incorporation of Ag@rGO nanosheets, the hydrogels possessed good conductivity. Pluronic F127 is a non-ionic block copolymer whose micelles remain non-ionized in aqueous media. Consequently, the incorporation of non-conductive PF127-QR micelles left the hydrogel conductivity unchanged, confirming that the micelles are seamlessly integrated without disrupting the pre-existing conductive Ag@rGO network. The strategy of incorporating Ag@rGO nanosheets into a polymer network could improve mechanical properties and conductivity. It was interesting that both SSA and SSA-QR hydrogels exhibited good self-healing behavior. Hydrogels were separated and reconnected for a few minutes (15 min), and the healed hydrogels could be observed via suspension using a tweezer (Figure 4e,f). SSA and SSA-QR exhibited self-healing capacity mainly due to ionic, hydrogen, and hydrophobic bonds within the hydrogels [39,40].

### 2.5. Adhesive Performance, Antibacterial Properties, and Antioxidant Activities

**Figure 5:**
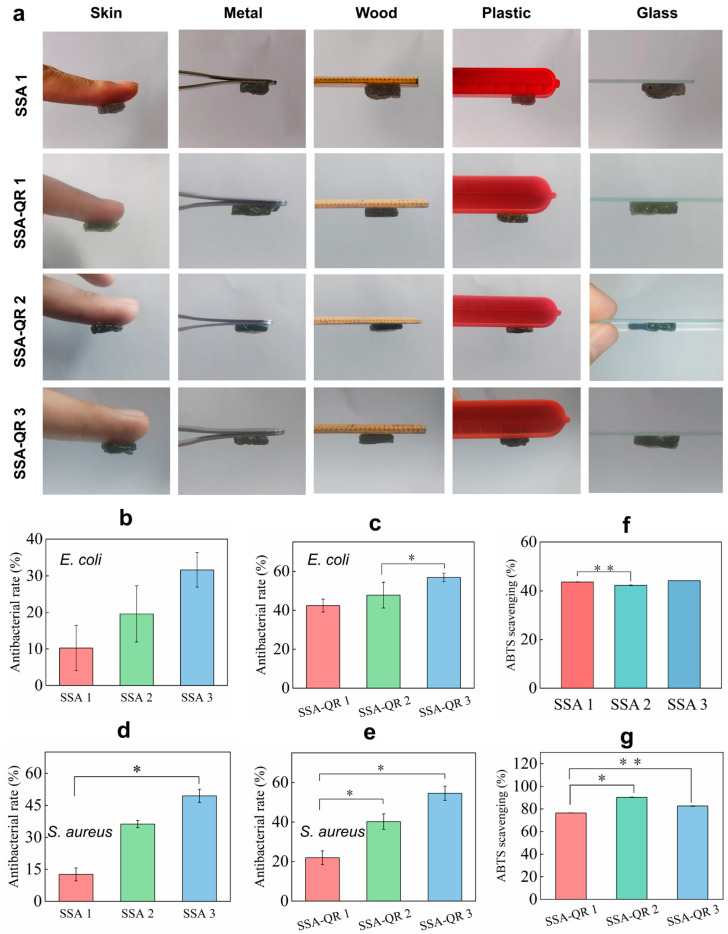
(**a**) The adhesive performance of SSA and SSA-QR hydrogels was evaluated by investigating their adhesion on the surface of different materials, including human skin, metal, wood, plastic, and glass. Noted that SSA-2 and SSA-3 hydrogels exhibited similar adhesive performance to SSA-1 hydrogel. The antibacterial ratios of SSA and SSA-QR hydrogels against (**b**,**c**) *E. coli* and (**d**,**e**) *S. aureus.* The ABTS scavenging abilities of (**f**) SSA and (**g**) SSA-QR hydrogels. * *p* < 0.05 and ** *p* < 0.01.

Adhesive performance was also an impressive property of hydrogels for wide biomedical applications, such as wound sealants, tissue-attached drug carriers, and so on [41,42]. The adhesive properties of SSA and SSA-QR hydrogels were macroscopically evaluated by adhering them to the surfaces of different materials, including skin, metal, wood, plastic, and glass (Figure 5a). Both hydrogels exhibited excellent adhesion to these materials. The adhesion mechanism of the SSA and SSA-QR hydrogels is one that several groups from SF, such as hydroxyl, carboxyl, and amino groups, could form the hydrogen bond on different substrate surfaces, and multivalent cationic groups from sodium alginate; also, the π-π interactions that occur can form metal coordination bonds with the metal matrix surface [43,44].

The antibacterial activities of SSA and SSA-QR hydrogels were investigated using the typical bacteria, Gram-positive *S. aureus* and Gram-negative *E. coli* (Figure 5b–e). With increasing the content of Ag@rGO nanosheets in SSA hydrogels, the antibacterial rates against *E. coli* and *S. aureus* increased gradually. Sample SSA 3 exhibited the highest antibacterial rates on both bacteria (Figure 5b,d). In our previous work, the strategy of incorporating Ag@rGO nanosheets into hydrogels was shown to be effective in improving the hydrogels’ antibacterial activity [29]. Here, it was observed that, upon incorporation of PF127-QR micelles into SSA hydrogels, antibacterial activity was further improved. It was also verified that SSA-QR hydrogels performed higher antibacterial rates than SSA hydrogels accordingly (Figure 5c,e). Quercetin has shown antibacterial activity against a wide range of bacterial strains and its antibacterial activity has been linked to its solubility, which further influences its interactions with the bacterial cell membrane [45,46]. Here, by incorporating Ag@rGO nanosheets and quercetin into hydrogels simultaneously, the antibacterial activity can be significantly improved.

The antioxidant activity of SSA and SSA-QR hydrogels was evaluated using ABTS, and ABTS scavenging rates were obtained in (Figure 5f,g). ABTS scavenging rates of SSA hydrogel were around 40%; SSA-QR hydrogels exhibited higher ABTS scavenging rates than SSA hydrogels. Sample SSA-QR 3 hydrogel had the highest ABTS scavenging rate (Figure 5g). Quercetin, as a natural polyphenol, is the most important dietary antioxidant. Quercetin can clear ROS and inhibit the production of cytokines (IL-4, IL-5, IL-9, and IL-13) by Th2 cells [47]. In addition, quercetin can neutralize ROS directly and inactivate several molecules with pro-oxidant capacity [48]. The incorporation of quercetin-loaded micelles into hydrogels could significantly enhance the antioxidant activity of the hybrid hydrogels.

### 2.6. Cytocompatibility, Hemocompatibility, and Cell Pro-Migration Ability Evaluation

**Figure 6:**
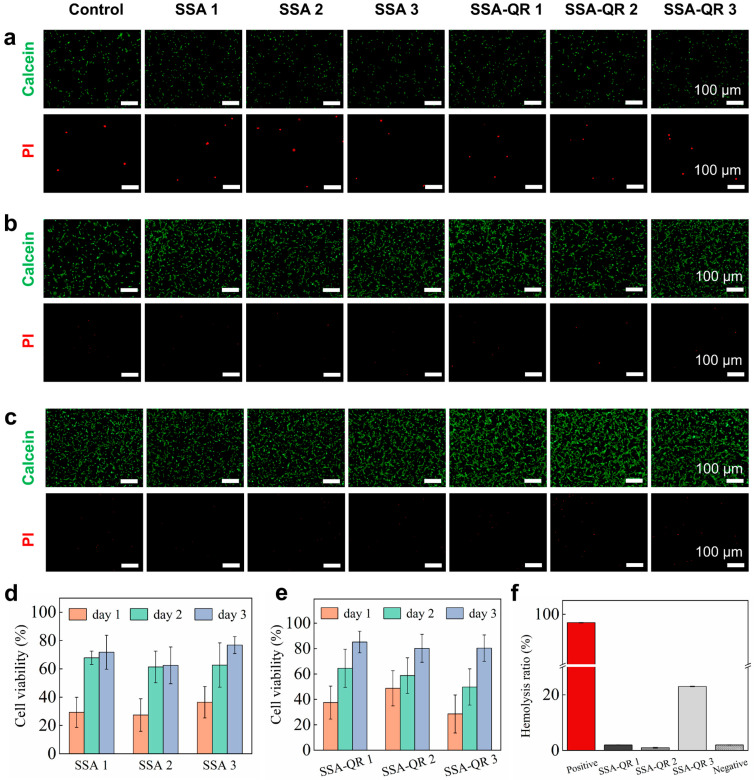
The fluorescent microscopy images of NIH 3T3 cells after culturing with SSA and SSA-QR hydrogel extracts at (**a**) day 1, (**b**) day 2, and (**c**) day 3. Calcein-AM/PI staining was used; green indicates the live cells and red indicates the dead cells. Cell viabilities of NIH 3T3 cells were obtained using CCK-8 assay after culturing with (**d**) SSA hydrogels extracts and (**e**) SSA-QR hydrogels extracts. (**f**) The hemolysis ratios of SSA-QR hydrogels.

The cytocompatibility of SSA and SSA-QR hydrogels was investigated by culturing with NIH 3T3 cells, and the cells’ morphology over days was observed using fluorescent microscopy images (Calcein-AM/PI staining) (Figure 6a–c); cell viability was measured by CCK-8 analysis (Figure 6d,e). From the fluorescent microscopy images, the number of cells apparently increased from day 1 to day 3. Especially on day 1, some dead cells (stained red) were observed, and on day 3, almost none were observed. The cell viability after culturing with SSA and SSA-QR hydrogel extracts increased over days. Due to the blood contact being inevitable for biomedical hydrogels in tissue engineering applications, the hemolysis rates of SSA-QR hydrogels were measured using the SD rat blood, as shown in Figure 6f. SSA-QR 1 and SSA-QR 2 exhibited excellent hemocompatibility, with hemolysis rates of 2.08 ± 0.02% and 1.05 ± 0.11%, both well below the 5% safety threshold. In sharp contrast, SSA-QR 3 induced substantial hemolysis (23.71 ± 0.07%), exceeding the acceptable limit for blood-contacting devices, 5% by the Food and Drug Administration (FDA). This deleterious, dose-dependent escalation is ascribed to the burst release of Ag^+^ ions and/or mechanical disruption of erythrocyte membranes by aggregated Ag@rGO nanosheets, underscoring the imperative for precise dosage optimization in downstream applications.

**Figure 7:**
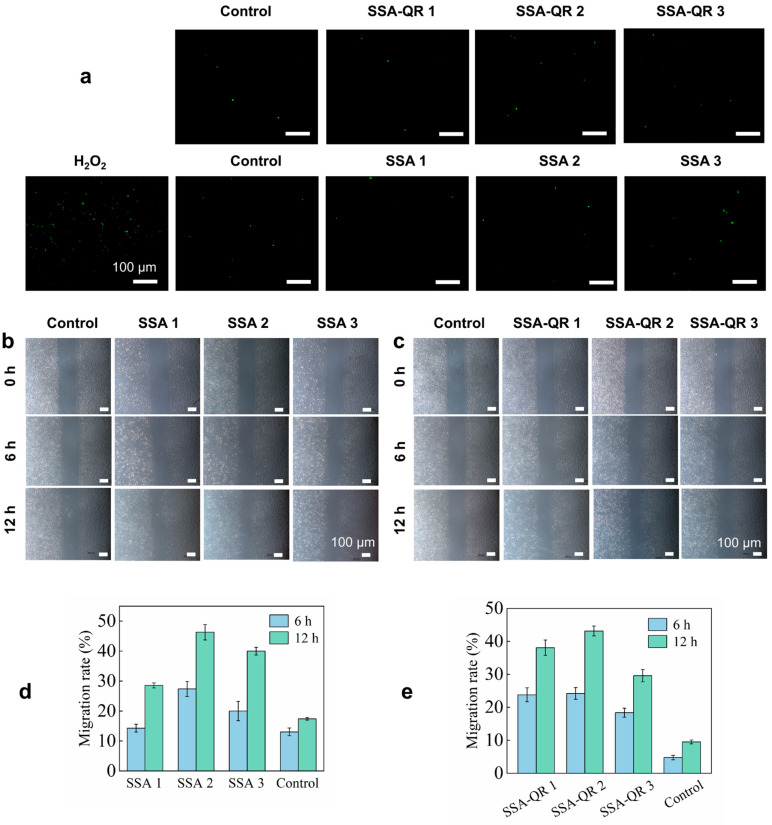
(**a**) The SSA and SSA-QR hydrogels’ influence on the intracellular ROS levels in NIH 3T3 cells. The cells were treated with H_2_O_2_ and the DCFH-DA staining approach was employed. The cell pro-migration abilities facilitated by SSA and SSA-QR hydrogels were investigated using the scratch assay. The optical images of NIH 3T3 cells after culturing with (**b**) SSA and (**c**) SSA-QR hydrogel extracts for 6 and 12 h. (**d**,**e**) The calculated migration rates of NIH 3T3 cells.

In addition, the intracellular ROS levels of NIH 3T3 cells exposed to SSA and SSA-QR hydrogels were evaluated; H_2_O_2_ was used as a stimulator and 2′,7′-dichlorodihydrofluorescein diacetate (DCFH-DA) staining was employed. The fluorescent images are shown in Figure 7a. The groups treated with SSA and SSA-QR hydrogel extracts exhibited a significant decrease compared to the H_2_O_2_-treated group; however, they displayed the same intracellular ROS level as the control group (without H_2_O_2_ treatment).

The cell scratch assay was used to investigate the cell pro-migration ability of SSA and SSA-QR hydrogels (Figure 7b,c). The scratch lines were clearly visible at 0 h. After culturing, some cells crossed the scratch lines and migrated into the blank areas. At hour 12, the interfaces between cell-growing areas and blank areas became indistinct, and more cells grew and proliferated in the blank areas. Furthermore, migration rates were statistically calculated using ImageJ, and the results are shown in Figure 7d,e. Sample SSA 2 hydrogel exhibited a higher migration rate than SSA 1 and SSA 3 hydrogels, and SSA-QR 2 hydrogel displayed a higher migration rate than SSA-QR 1 and SSA-QR 3 hydrogels. Silk fibroin/alginate hydrogels have demonstrated excellent cytocompatibility and cell pro-migration ability and have been used in cell-laden hydrogels or bioinks for a wide range of biomedical applications, such as bone tissue engineering, myocardial infarction, wound dressing, etc. [49,50,51]. For SSA hydrogels only incorporating Ag@rGO nanosheets, SSA 3 hydrogel exhibited the highest cell viability, and SSA 2 hydrogel demonstrated the highest cell migration rate, indicating that the incorporation of Ag@rGO nanosheets could further improve the cytocompatibility of silk fibroin/alginate hydrogels; however, the balance of cytocompatibility and cell pro-migration ability needs to be explored. For SSA-QR hydrogels incorporating Ag@rGO nanosheets and PF127-QR micelles, SSA-QR 2 displayed the best cell viability and cell pro-migration ability, indicating that a suitable content of quercetin has a positive effect on cytocompatibility and cell pro-migration ability.

## 3. Materials and Methods

### 3.1. Chemicals

Quercetin (97%), cocoon, calcium chloride (CaCl_2_), sodium carbonate (Na_2_CO_3_), lithium bromide (LiBr), silver nitrate (AgNO_3_), polyether-F127 (PF-127), sodium alginate (SA), potassium persulfate (K_2_S_2_O_2_), graphene oxide (GO), polyvinylpyrrolidone (average molecular weight 8000 Da), l-ascorbic acid (L-AA, ≥99.7%), and 2,2-Azion-bis(3-ethylbenzothiazoline-6-sulfonic acid) diammonium salt (ABTS) were purchased from Macklin Co., Ltd. (Shanghai, China). Calcein-AM/PI cell activity and cytotoxicity detection kit was purchased from Biyuntian Biotechnology Co., Ltd. (Shanghai, China). Millipore water was used in this work, and Millipore water was prepared using a Milli-Q50 SP Reagent Water System (Millipore Corporation, Billerica, MA, USA). All purchased chemicals and solvents were used directly without further purification.

### 3.2. Preparation of Silver-Doped Reduced Graphene Oxide (Ag@rGO) Nanosheets

Ag@rGO nanosheets were facilely prepared according to a previous report with minor modifications [52]. Briefly, 0.4 g of L-ascorbic acid, 0.1 g of polyvinylpyrrolidone, and 0.1 g of silver nitrate were mixed, and 0.1 mL of graphene oxide aqueous solution (2 mg/mL) was mixed and treated using ultrasonication for 20 min. Then, the mixed solution was stirred (700 rpm) at 80 °C for 2 h. After that, the reaction solution was dialyzed for 3 days and lyophilized to obtain the final Ag@rGO powder.

### 3.3. Preparation of Quercetin Loaded with PF127 (PF127-QR) Micelles

PF127-QR micelles were fabricated based on previous reports [27,28]. Quercetin and PF-127 polymers were dissolved in methanol at a quercetin/PF-127 weight ratio of 1:90 and the mixture was stirred gently. Subsequently, the mixture was concentrated using a rotary evaporator (40 °C). Then, the product was rehydrated in deionized water and stirred (500 rpm) to form micelles via self-assembly. With further extraction and filtration, the unencapsulated quercetin was removed. Finally, the yellow PF127-QR powder was obtained using freeze-drying and stored at −20 °C for subsequent use [53].

### 3.4. Fabrication of Hybrid Hydrogels

First, silk fibroin was prepared from the mulberry silkworm based on previous reports [54]. In brief, 8 g of silkworm cocoons were cut into small pieces. The obtained pieces were boiled in 400 mL of Na_2_CO_3_ solution (0.05 M) for 30 min to remove the sesame protein from the silkworm. Next, the boiled silkworm was washed 3 times using Millipore water, and the white product was obtained. Then, the washed, degummed silk was spread flat on tin foil and placed in an oven (60 °C) to dry completely. Next, the dried, degummed silk was dissolved in 20 mL of LiBr solution (9.5 M). The obtained solution was dialyzed against Millipore water for 72 h, with the water changed 3 times a day. Then, the resulting solution was incubated in polyethylene glycol for 6 h to obtain a silk fibroin solution, and the concentration was measured at 8 wt%. The obtained SF solution was stored at 4 °C for later use.

Before fabricating hydrogels, alginate solution (2 wt%) and CaCl_2_ solution (2 wt%) were prepared, respectively. At room temperature, according to the designed ratio (Table 1), SF solution, alginate solution, and Ag@rGO nanosheets were mixed to obtain the mixed solution. Then, the CaCl_2_ solution (0.05 M) was added dropwise to SSA hydrogels. For each SSA hydrogel, 50 mg PF127-QR was introduced to obtain SSA-QR hydrogels, as shown in Table 2.

### 3.5. Fourier Transform Infrared Spectroscopy (FT-IR) Evaluation

The chemical interactions between SF, alginate, and Ag@rGO nanosheets in SSA and SSA-QR hydrogels were evaluated using an infrared spectrometer of Fourier transform (FT-IR, ThermoFisher Nicolelis5, Bruker, Bremen, Germany). The tested sample was pressed into a thin layer, and the FT-IR spectrum was collected in the range of 4000–500 cm^−1^ using an attenuated total reflectance (ATR) mode. The resolution is 4 cm^−1^ with an average of 64 scans per spectrum [55].

### 3.6. Scanning Electron Microscopy (SEM) Investigation

The morphology and porous microstructure of the fabricated SSA and SSA-QR hydrogel were observed using a field-emission scanning electron microscope (SEM, Hitachi Regulus 8220, Tokyo, Japan) [52]. The lyophilized hydrogels were used, and the slices were obtained after immersing them in liquid nitrogen. Prior to observation, the sample was sprayed with a thin platinum (120 s spraying). Then, SEM images were obtained accordingly. In addition, the morphology of Ag@rGO nanosheets was investigated using SEM, and element-mapping images were obtained.

### 3.7. Transmission Electron Microscopy (TEM) Investigation

The morphology of Ag@rGO nanosheets and PF127-QR micelles was investigated using transmission electron microscopy (TEM, Tecnai G2 F20, FEI, Hillsboro, OR, USA) [56]. The tested samples were dispersed in Millipore water and treated using sonication for 2 h to ensure uniform dispersion. Then, the copper grid was immersed in the sonicated solution for 5 s, removed, and dried. The TEM images were captured accordingly, and element-mapping images were also obtained.

### 3.8. X-Ray Photoelectron Spectroscopy (XPS)

The silver nanoparticles doped on rGO nanosheets were evaluated using X-ray photoelectron spectroscopy (XPS, Thermo Scientific, Waltham, MA, USA). The surface of the tested sample was irradiated with an energy resolution of 0.05 electron volts to obtain the XPS spectrum. According to the XPS spectrum, the elemental composition and chemical state of Ag@rGO nanosheets were analyzed by combining energy positions and peak areas [52].

### 3.9. Dynamic Light Scattering (DLS)

The diameter distribution of PF127-QR micelles was investigated using dynamic light scattering (DLS, Malvern Zetasizer 3000E, Warriewood, Australia) [57]. The tested PF127-QR micelles were dissolved in Millipore water (approximately 1 mg/mL) and fully dispersed by ultrasonication. Then, the solution was further filtered to remove large impurity particles for the DLS test. The size distribution of PF127-QR micelles was recorded.

### 3.10. Encapsulation Efficiency (EE) and Drug Loading (LC)

The encapsulation efficiency (*EE*) and drug loading (*LC*) of quercetin in PF127-QR were investigated [28]. The absorbance of quercetin-methanol-aqueous solution was determined at 256 nm by ultraviolet–visible spectrophotometry (UV–vis), and a standard curve was obtained using the quercetin concentration range of 1–50 μg/mL [28]. Subsequently, the concentration of PF127-QR in the same solvent system was determined based on the standard curve, and the following Equations (1) and (2) were used to calculate *EE* and *LC*, respectively:

(1) $$EE\;(\%)\;=\;\frac{W_{QR}}{W_{0}}\;\times \;100\%$$

(2) $$LC\;(\%)=\frac{W_{QR}}{W_{P}}\;\times \;100\%$$

where *W_QR_* indicates the weight of quercetin, *W*_0_ means the weight of quercetin initially fed for preparing PF127-QR micelles, and *W_P_* is the net weight of quercetin in the product of PF127-QR micelles.

### 3.11. Drug Release of Quercetin in SSA-QR Hydrogel

According to the standard curve from the last subsection, the release profiles of quercetin from composite hydrogels were studied [58]. Briefly, 1 mL of the composite hydrogel was immersed in 3 mL of phosphate-buffered saline (PBS, pH 7.2). Then, at a predetermined time interval, 1 mL of liquid was taken for UV–vis spectrophotometer analysis (256 nm). Then, 1 mL of fresh PBS was added to the hydrogel solution to maintain a constant volume. The release concentration of quercetin from the composite hydrogel was analyzed using a quercetin standard curve. The quercetin testing was accomplished at 37 °C with oscillating at 120 rpm/min.

### 3.12. Compression Testing

The compressive strength of SSA and SSA-QR composite hydrogels was evaluated by using a universal mechanical testing machine under 80% compression [59] (CMT-4103; Zhuhai SUST Instrument Co., Ltd., Zhuhai, China). The tested hydrogels were prepared into a cylindrical sample with a height of 10 mm and a diameter of 10 mm for the compressive test.

### 3.13. Dynamic Swelling Properties of Hydrogels

The dynamic swelling rates of the hydrogel were determined by the weight method [60]. After freeze-drying, the hydrogel was trimmed into regular shapes and weighed (*M_A_*). The resulting hydrogel was immersed in Millipore water, and the surface water was absorbed and weighed (*M_B_*) at intervals until the hydrogel had a constant weight. The swelling rate of the hydrogel is calculated by the following Equation (3):

(3) $$Swelling\;rate\;(\%)\;=\;\frac{M_{B}-M_{A}}{M_{A}}\times 100\%$$

where *M_A_* is the dry weight of the hydrogel, *M_B_* is the constant weight of the hydrogel after immersing in Millipore water, and each sample is repeated in three groups.

### 3.14. Conductivity Test

The conductivity of SSA and SSA-QR hydrogels was tested with a digital quad probe tester (RT-9) [61]. During measurement, the probe was placed in a wet hydrogel, and the current intensity was set to 10 mA to measure conductivity. In addition, a self-made circuit was used to measure macroscopic conductivity.

### 3.15. Self-Healing Ability

The self-healing ability of SSA and SSA-QR hydrogels can be directly evaluated by cutting and reconnecting hydrogels [62]. Briefly, the hydrogels were cut and placed together for 10 min. Although there were relatively obvious cracks on the surface of the hydrogel, it was able to maintain its original shape without external force.

### 3.16. Adhesion Performance

Macro adhesion experiment: The hydrogel is prepared into a block of 1 cm × 1 cm (length × width), and the adhesion ability of the hydrogel to different materials is evaluated by the sticking effect of the hydrogel with skin, tweezers, wooden rulers, plastic clips, glass slides, etc. [63].

### 3.17. Antioxidant Activity

Antioxidant tests of SSA, SSA-QR hydrogels were evaluated using 2,2′-Azinobis-(3-ethylbenzthiazoline-6-sulphonate (ABTS) radical scavenging activity test [58]. Mix the ABTS and potassium persulfate solutions in equal volumes and incubate for 12 h in the dark. Then, the mixed solution was diluted 25 times and then used. Add 0.02 g of lyophilized hydrogel to 5 mL of diluted solution, incubate away from light for 0.5 h, measure absorbance at 734 nm wavelength using an ultraviolet–visible spectrophotometer, and calculate the clearance using the following Equation (4):

(4) $$ABTS\;clearance\;rate\;(\%)\;=\frac{A_{0}-A_{1}}{A_{0}}\times 100\%$$

where *A*_0_ and *A*_1_ are the absorption values of the blank group and the sample group, respectively.

### 3.18. Hemolysis Evaluation

Take the fresh blood of the mice and add an appropriate amount of 0.9% normal saline to prepare a red blood cell suspension. Weigh 2.5 mg of dry hydrogel into a 1.5 mL centrifuge tube, add 1 mL 0.9% normal saline, incubate for 30 min at 37 °C, then add 20 μL red cell suspension to the centrifuge tube, add 1 mL of normal saline and 20 μL of red cell suspension in the negative control group, add 1 mL of ultrapure water and 20 μL of red cell suspension in the positive control group, and incubate with the sample group at 37 °C for 1 h. After centrifuging (3000 rpm, 5 min), collect the supernatant and measure its absorbance at 545 nm [64]. The hemolysis rate is calculated using the following Equation (5):

(5) $$Hemolysis\;rate\;(\%)\;=\frac{A_{s}-A_{n}}{A_{p}-A_{n}}\times 100\%$$

where *A_s_*, *A_p_*, and *A_n_* are the absorbance values corresponding to the sample group, the positive control group, and the negative control group, respectively.

### 3.19. Antibacterial Activity

The antibacterial properties of hydrogels were evaluated using liquid antibacterial treatment to assess their activity against Gram-negative *Escherichia coli* (*E. coli*, ATCC 25922) and Gram-positive *Staphylococcus aureus* (*S. aureus*, ATCC 6538). A total of 0.015 g of lyophilized hydrogel was added to a glass test tube containing 100 μL of LB medium and a bacterial suspension (10^7^ CFU mL^−1^) and the mixture was incubated at 37 °C for 12 h, with three parallel samples per group. Finally, the absorbance of the mixture at 600 nm was measured. Test tubes with no hydrogel samples were added as the control group [58]. The antibacterial rate is calculated by the following Equation (6):

(6) $$Antibacterial\;ratio\;(\%)\;=\frac{K_{0}-K_{1}}{K_{0}}\times 100\%$$

where *K*_0_ and *K*_1_ are the absorbance values of the control group and the sample group, respectively. The test was repeated three times per group.

### 3.20. Biocompatibility

In vitro cytocompatibility assay of hydrogels: The lyophilized hydrogel was cut into small pieces, placed in a centrifuge tube, and sterilized with ultraviolet (UV) radiation for 4 h. Prepare a hydrogel extract (5 mg/mL) in Dulbecco’ modified eagle medium (DMEM) and incubate overnight at 37 °C. Then, mouse embryonic fibroblasts (NIH-3T3 cells, ATCC, CRL-1658^TM^, Manassas, WV, USA) were inoculated on a 96-well plate at 5000 cells per well. The sample group, control group, and blank group were cultured separately with 200 µL of medium per well and incubated at 37 °C in a carbon dioxide incubator for 1, 2, and 3 days, respectively. The cell counting kit-8 (CCK-8) was then used for detection, and absorbance at 450 nm (*OD*_450_) was measured using a microplate reader [28]. Cell viability is calculated by the following Equation (7):

(7) $$Cell\;Viability\;(\%)\;=\;\frac{A_{b}-A_{0}}{A_{c}-A_{0}}\times 100\%$$

where *A_b_*, *A_c_*_,_ and *A*_0_ represent absorbance values of the sample group, the control group, and the blank group, respectively.

### 3.21. Cell Scratch Assay

Scratch experiments were used to observe cell migration. The culture plug-in was placed on a six-well plate. The cells were inoculated into the ibidi culture plug-in for 24 h (cell density: 5 × 10^4^ cells per well), and the plug-in was removed. A total of 1 mL of fresh hydrogel extract was added to the sample group, 1 mL of fresh culture medium was added to the blank group, and the scratch widths of 0 h, 6 h, and 12 h were recorded, respectively [62]. Cell migration was observed using an inverted microscope, and cell mobility was analyzed using ImageJ (Version 1.53). The mobility calculation formula is as follows (8):

(8) $$Migration\;Rate\;(\%)\;=\;\frac{S_{0}-S_{n}}{S_{0}}\times 100\%$$

where *S*_0_ represents the initial scratch width and *S_n_* represents the width of the scratch at 6 h and 12 h.

SSA-QR and SSA hydrogels were stained with a Calcein acetoxymethyl ester/propidium iodide (Calcein-AM/PI) cell activity and cytotoxicity assay kit (Biyuntian, Shanghai, China) [65]. The cells were seeded in a 35 mm culture dish at 500,000 cells per well. The sample group was treated with the extract solution, and the control group was treated with the culture solution and cultured for 24 h. Stain the cells according to the instructions and obtain fluorescence images using a fluorescence microscope (Nikon, Kokyo, Japan).

### 3.22. Statistical Analysis

The experimental results were presented in the form of mean ± standard deviation (SD). Univariate or two-factor repeated measures ANOVA and graph basis tests were conducted using SPSS software Version 26.0.0.0 (SPSS Inc., Chicago, IL, USA) with statistical significance defined according to *p*-values of <0.05, <0.01, and <0.001, which represent 95%, 99%, and 99.9% confidence, respectively.

## 4. Conclusions

In summary, we have prepared interconnected porous hybrid hydrogels comprising silk fibroin, alginate, silver-doped rGO nanosheets, and quercetin-encapsulated PF-127 micelles. The mechanical strength, equilibrium swelling rate, and quercetin release profiles could be regulated by adjusting the silk fibroin and Ag@rGO nanosheet contents. The prepared hydrogels, with or without PF127-QR micelles, exhibited conductivity, self-healing, and strong adhesion to various matrix surfaces, including skin, metal, wood, plastic, and glass. Additionally, the incorporation of quercetin-loaded PF-127 micelles increased the hydrogels’ antioxidant and antibacterial activities compared to those without quercetin loading. The hybrid hydrogels with a low content of Ag@rGO nanosheets displayed good hemocompatibility, and increasing the Ag@rGO nanosheet content led to a significant increase in the hemolysis ratio of the hydrogels. Furthermore, the fabricated hydrogels demonstrated intracellular ROS scavenging, excellent cytocompatibility, and cell-promoting migration. The obtained data confirmed that the designed hybrid hydrogels have potential for biomedicine and tissue regeneration. The specific biomedical application of the fabricated hydrogels needs in vitro and in vivo confirmation in the near future.

## Figures and Tables

**Table 1:** The composition of SF, alginate, and Ag@rGO nanosheets to fabricate SSA hydrogel.

Hydrogels	SF Solution ^a^	Alginate Solution ^b^	Ag@rGO Nanosheets	CaCl_2_ Solution ^c^
SSA 1	0.8 mL	10 mL	3 mg	1.2 mL
SSA 2	1.25 mL	10 mL	5 mg	1.2 mL
SSA 3	2.5 mL	10 mL	10 mg	1.2 mL

^a^ SF solution: 8 wt%. ^b^ alginate solution: 2 wt%. ^c^ CaCl_2_ solution: 0.05 M.

**Table 2:** The composition of SF, alginate, Ag@rGO nanosheets, and PF127-QR micelles to fabricate SSA-QR hydrogel.

Hydrogels	SF Solution ^a^	Alginate Solution ^b^	Ag@rGO Nanosheets	PF127-QR Micelles	CaCl_2_ Solution ^c^
SSA-QR 1	0.8 mL	10 mL	3 mg	50 mg	1.2 mL
SSA-QR 2	1.25 mL	10 mL	5 mg	50 mg	1.2 mL
SSA-QR 3	2.5 mL	10 mL	10 mg	50 mg	1.2 mL

^a^ SF solution: 8 wt%. ^b^ alginate solution: 2 wt%. ^c^ CaCl_2_ solution: 0.05 M.

## Data Availability

Data will be made available on request.

## References

[1] Segneanu A.-E., Bejenaru L.E., Bejenaru C., Blendea A., Mogoşanu G.D., Biţă A., Boia E.R. (2025). Advancements in hydrogels: A comprehensive review of natural and synthetic innovations for biomedical applications. Polymers.

[2] Lu P., Ruan D., Huang M., Tian M., Zhu K., Gan Z., Xiao Z. (2024). Harnessing the potential of hydrogels for advanced therapeutic applications: Current achievements and future directions. Signal Transduct. Target. Ther..

[3] Zoeller K., To D., Bernkop-Schnuerch A. (2025). Biomedical applications of functional hydrogels: Innovative developments, relevant clinical trials and advanced products. Biomaterials.

[4] Kaczmarek B., Nadolna K., Owczarek A. (2020). The physical and chemical properties of hydrogels based on natural polymers. Hydrogels Based on Natural Polymers.

[5] Vasile C., Pamfil D., Stoleru E., Baican M. (2020). New Developments in Medical Applications of Hybrid Hydrogels Containing Natural Polymers. Molecules.

[6] Li Z., Lin Z. (2021). Recent advances in polysaccharide-based hydrogels for synthesis and applications. Aggregate.

[7] Sharma S., Bhende M., Goel A. (2024). A review: Polysaccharide-based hydrogels and their biomedical applications. Polym. Bull..

[8] Tang Y., Zhang X., Li X., Ma C., Chu X., Wang L., Xu W. (2022). A review on recent advances of Protein-Polymer hydrogels. Eur. Polym. J..

[9] Zheng H., Zuo B. (2021). Functional silk fibroin hydrogels: Preparation, properties and applications. J. Mater. Chem. B.

[10] Lin D., Li M., Wang L., Cheng J., Yang Y., Wang H., Ye J., Liu Y. (2024). Multifunctional Hydrogel Based on Silk Fibroin Promotes Tissue Repair and Regeneration. Adv. Funct. Mater..

[11] Fernández-González A., de Lorenzo González C., Rodríguez-Varillas S., Badía-Laíño R. (2024). Bioactive silk fibroin hydrogels: Unraveling the potential for biomedical engineering. Int. J. Biol. Macromol..

[12] Peng L., Zhou P., Liao F., Liu G., Bao S., Yang X., Xiao B., Duan L. (2025). Silk fibroin hydrogels: Gelation mechanisms, fabrication techniques, and biomedical applications. Int. J. Biol. Macromol..

[13] De Giorgio G., Matera B., Vurro D., Manfredi E., Galstyan V., Tarabella G., Ghezzi B., D’Angelo P. (2024). Silk Fibroin Materials: Biomedical Applications and Perspectives. Bioengineering.

[14] Ren Y., Wang Q., Xu W., Yang M., Guo W., He S., Liu W. (2024). Alginate-based hydrogels mediated biomedical applications: A review. Int. J. Biol. Macromol..

[15] Abka-Khajouei R., Tounsi L., Shahabi N., Patel A.K., Abdelkafi S., Michaud P. (2022). Structures, properties and applications of alginates. Mar. Drugs.

[16] Chen X., Wu T., Bu Y., Yan H., Lin Q. (2024). Fabrication and Biomedical Application of Alginate Composite Hydrogels in Bone Tissue Engineering: A Review. Int. J. Mol. Sci..

[17] Vaziri A.S., Vasheghani-Farahani E., Hosseinzadeh S., Bagheri F., Büchner M., Schubert D.W., Boccaccini A.R. (2024). Genipin-Cross-Linked Silk Fibroin/Alginate Dialdehyde Hydrogel with Tunable Gelation Kinetics, Degradability, and Mechanical Properties: A Potential Candidate for Tissue Regeneration. Biomacromolecules.

[18] Compaan A.M., Christensen K., Huang Y. (2017). Inkjet Bioprinting of 3D Silk Fibroin Cellular Constructs Using Sacrificial Alginate. ACS Biomater. Sci. Eng..

[19] Nguyễn T.T., Ratanavaraporn J., Yodmuang S. Alginate-silk fibroin Bioink: A printable hydrogel for tissue engineering. Proceedings of the 2019 12th Biomedical Engineering International Conference (BMEiCON).

[20] du Preez H.N., Halma M. (2024). Graphene-based nanomaterials: Uses, environmental fate, and human health hazards. Nano Biomed. Eng..

[21] Gong D., Zhai Q., Wu M., Tao X., Wang F. (2025). Promotion of rat femoral distal bone defect repair using alginate-silk fibroin composite hydrogel. Int. Immunopharmacol..

[22] Yang S., Wang F., Zhao H., Zhang Y., He W., Wang P., Fu C., Chen W., Zhu Y., Zhang Y. (2026). Graphene oxide/sodium alginate/silk fibroin extracellular matrix-like sponge for acellular and growth factor-free bone regeneration. Carbohydr. Polym..

[23] Xue P., Hu X., Chang E., Wang L., Chen M., Wu T.-H., Lee D.-J., Foster B.L., Tseng H.C., Ko C.-C. (2021). Deficiency of optineurin enhances osteoclast differentiation by attenuating the NRF2-mediated antioxidant response. Exp. Mol. Med..

[24] Hu B., Ouyang Y., Zhao T., Wang Z., Yan Q., Qian Q., Wang W., Wang S. (2024). Antioxidant Hydrogels: Antioxidant Mechanisms, Design Strategies, and Applications in the Treatment of Oxidative Stress-Related Diseases. Adv. Healthc. Mater..

[25] Singh P., Arif Y., Bajguz A., Hayat S. (2021). The role of quercetin in plants. Plant Physiol. Biochem..

[26] Yi Y., Yang Z., Zhou C., Yang Y., Wu Y., Zhang Q. (2024). Quercetin-encapsulated GelMa hydrogel microneedle reduces oxidative stress and facilitates wound healing. Nano TransMed.

[27] Nie L., Ding X., Zhao Y., Ding P., Zhao M., Sun Y., Sun Y., Jiang G. (2025). L-hydroxyproline-conjugated chitosan based hydrogel integrated with curcumin-loaded PF127 micelles to promote wound healing. Int. J. Biol. Macromol..

[28] Ding P., Lu Y., Zhao C., Guo W., Nie L. (2024). Preparation of injectable self-healing hydrogels using carboxymethyl chitosan and oxidized dextran with incorporating quercetin-loaded PF127 micelles for wound healing. Mater. Today Commun..

[29] Guo W., Huang Y., Lu Y., Ding X., Ding P., Okoro O.V., Shavandi A., Nie L. (2025). Adhesive, self-healing, injectable, conductive, and antibacterial hydrogels based on oxidized dextran and carboxymethyl chitosan incorporating silver nanoparticles doped rGO nanosheets. Mater. Today Commun..

[30] İspir E., İnal M., Gün Gök Z., Yiğitoğlu M. (2024). Synthesis, characterization and in vitro release analysis of pluronic F127 copolymer micelles containing quercetin as a hydrophobic drug. Polym. Bull..

[31] Ahamed M., Akhtar M.J., Khan M.M., Alhadlaq H.A. (2021). A novel green preparation of Ag/RGO nanocomposites with highly effective anticancer performance. Polymers.

[32] Vasconcelos A., Freddi G., Cavaco-Paulo A. (2008). Biodegradable Materials Based on Silk Fibroin and Keratin. Biomacromolecules.

[33] Lu Q., Zhang B., Li M., Zuo B., Kaplan D.L., Huang Y., Zhu H. (2011). Degradation Mechanism and Control of Silk Fibroin. Biomacromolecules.

[34] Dalal S.R., Hussein M.H., El-Naggar N.E.-A., Mostafa S.I., Shaaban-Dessuuki S.A. (2021). Characterization of alginate extracted from Sargassum latifolium and its use in Chlorella vulgaris growth promotion and riboflavin drug delivery. Sci. Rep..

[35] Flórez-Fernández N., Domínguez H., Torres M.D. (2019). A green approach for alginate extraction from Sargassum muticum brown seaweed using ultrasound-assisted technique. Int. J. Biol. Macromol..

[36] Daemi H., Barikani M. (2012). Synthesis and characterization of calcium alginate nanoparticles, sodium homopolymannuronate salt and its calcium nanoparticles. Sci. Iran..

[37] Davarnejad R., Layeghy K., Soleymani M., Ayazi A. (2022). Encapsulation of Quercetin in a Mixed Nanomicellar System to Enhance its Cytotoxicity against Breast Cancer Cells. Chem. Eng. Technol..

[38] Liang Y., Qiao L., Qiao B., Guo B. (2023). Conductive hydrogels for tissue repair. Chem. Sci..

[39] Devi V. K. A., Shyam R., Palaniappan A., Jaiswal A.K., Oh T.-H., Nathanael A.J. (2021). Self-Healing Hydrogels: Preparation, Mechanism and Advancement in Biomedical Applications. Polymers.

[40] Quan L., Xin Y., Wu X., Ao Q. (2022). Mechanism of Self-Healing Hydrogels and Application in Tissue Engineering. Polymers.

[41] Tang S., Feng K., Yang R., Cheng Y., Chen M., Zhang H., Shi N., Wei Z., Ren H., Ma Y. (2025). Multifunctional Adhesive Hydrogels: From Design to Biomedical Applications. Adv. Healthc. Mater..

[42] Xie C., Wang X., He H., Ding Y., Lu X. (2020). Mussel-Inspired Hydrogels for Self-Adhesive Bioelectronics. Adv. Funct. Mater..

[43] Zheng H., Lin N., He Y., Zuo B. (2021). Self-Healing, Self-Adhesive Silk Fibroin Conductive Hydrogel as a Flexible Strain Sensor. ACS Appl. Mater. Interfaces.

[44] Huang G., Tang Z., Peng S., Zhang P., Sun T., Wei W., Zeng L., Guo H., Guo H., Meng G. (2022). Modification of Hydrophobic Hydrogels into a Strongly Adhesive and Tough Hydrogel by Electrostatic Interaction. Macromolecules.

[45] Nguyen T.L.A., Bhattacharya D. (2022). Antimicrobial Activity of Quercetin: An Approach to Its Mechanistic Principle. Molecules.

[46] Zhao X., Cui X., Yang Y., Zhu L., Li L., Kong X. (2022). Synergistic Effect of Quercetin on Antibacterial Activity of Florfenicol Against Aeromonas hydrophila In Vitro and In Vivo. Antibiotics.

[47] Liu C., Cheng X., Wu Y., Xu W., Xia H., Jia R., Liu Y., Shen S., Xu Y., Cheng Z. (2023). Antioxidant Activity of Quercetin-Containing Liposomes-in-Gel and Its Effect on Prevention and Treatment of Cutaneous Eczema. Pharmaceuticals.

[48] Remigante A., Spinelli S., Straface E., Gambardella L., Caruso D., Falliti G., Dossena S., Marino A., Morabito R. (2022). Antioxidant Activity of Quercetin in a H_2_O_2_-Induced Oxidative Stress Model in Red Blood Cells: Functional Role of Band 3 Protein. Int. J. Mol. Sci..

[49] Ghorbani M., Vasheghani-Farahani E., Azarpira N., Hashemi-Najafabadi S., Ghasemi A. (2023). Dual-crosslinked in-situ forming alginate/silk fibroin hydrogel with potential for bone tissue engineering. Biomater. Adv..

[50] Ni Y., Hua Y., He Z., Hu W., Chen Z., Wang D., Li X., Sun Y., Jiang G. (2024). Release of exosomes from injectable silk fibroin and alginate composite hydrogel for treatment of myocardial infarction. J. Biomater. Appl..

[51] Rezaei F., Damoogh S., Reis R.L., Kundu S.C., Mottaghitalab F., Farokhi M. (2020). Dual drug delivery system based on pH-sensitive silk fibroin/alginate nanoparticles entrapped in PNIPAM hydrogel for treating severe infected burn wound. Biofabrication.

[52] Sudapalli A.M., Shimpi N.G. (2023). Ag@rGO coral reef morphology exhibits excellent optical properties and photocatalytic activity against methyl orange under sunlight. Diam. Relat. Mater..

[53] Lupu A., Rosca I., Gradinaru V.R., Bercea M. (2023). Temperature Induced Gelation and Antimicrobial Properties of Pluronic F127 Based Systems. Polymers.

[54] Jing Y., Ruan L., Jiang G., Nie L., Shavandi A., Sun Y., Xu J., Shao X., Zhu J. (2023). Regenerated silk fibroin and alginate composite hydrogel dressings loaded with curcumin nanoparticles for bacterial-infected wound closure. Biomater. Adv..

[55] Chen M., Wei L., Zhang W., Wang C., Xiao C. (2021). Fabrication and catalytic performance of a novel tubular PMIA/Ag@RGO nanocomposite nanofiber membrane. RSC Adv..

[56] Deng J., Li J., Yan L., Guo W., Ding X., Ding P., Liu S., Sun Y., Jiang G., Okoro O.V. (2024). Accelerated, injectable, self-healing, scarless wound dressings using rGO reinforced dextran/chitosan hydrogels incorporated with PDA-loaded asiaticoside. Int. J. Biol. Macromol..

[57] Patra A., Satpathy S., Shenoy A., Bush J., Kazi M., Hussain M.D. (2018). Formulation and evaluation of mixed polymeric micelles of quercetin for treatment of breast, ovarian, and multidrug resistant cancers. Int. J. Nanomed..

[58] Yan S., Xu S., Wang Y., You J., Guo C., Wu X. (2024). A Hydrogel Dressing Comprised of Silk Fibroin, Ag Nanoparticles, and Reduced Graphene Oxide for NIR Photothermal-Enhanced Antibacterial Efficiency and Skin Regeneration. Adv. Healthc. Mater..

[59] Wang Y., Niu C., Shi J., Yu W., Zhu C., Zhang Q., Mizuno M. (2021). A Carbodiimide Cross-Linked Silk Fibroin/Sodium Alginate Composite Hydrogel with Tunable Properties for Sustained Drug Delivery. Macromol. Mater. Eng..

[60] Ding P., Ding X., Li J., Guo W., Okoro O.V., Mirzaei M., Sun Y., Jiang G., Shavandi A., Nie L. (2024). Facile preparation of self-healing hydrogels based on chitosan and PVA with the incorporation of curcumin-loaded micelles for wound dressings. Biomed. Mater..

[61] Ruiz I., Castro S., Aedo V., Tapia M., González L., Aguayo C., Fernández K. (2024). Inclusion of Reduced Graphene Oxide to Silk Fibroin Hydrogels Improve the Conductive, Swelling and Wound Healing Capacity. ChemistrySelect.

[62] Li Y., Chen S., Zhang M., Ma X., Zhao J., Ji Y. (2024). Novel Injectable, Self-Healing, Long-Effective Bacteriostatic, and Healed-Promoting Hydrogel Wound Dressing and Controlled Drug Delivery Mechanisms. ACS Appl. Mater. Interfaces.

[63] Nezhad-Mokhtari P., Hamishehkar H., Farahpour M.R., Mehdipour A., Rahbarghazi R., Milani M., Mehrali M. (2024). Engineered bioadhesive Self-Healing nanocomposite hydrogel to fight infection and accelerate cutaneous wound healing. Chem. Eng. J..

[64] Liu G., Bao Z., Wu J. (2020). Injectable baicalin/F127 hydrogel with antioxidant activity for enhanced wound healing. Chin. Chem. Lett..

[65] Jiang X., Yang Z., Zhang J., Liang H., Wang H., Lu J. (2024). Preparation and characterization of photosensitive methacrylate-grafted sodium carboxymethyl cellulose as an injectable material to fabricate hydrogels for biomedical applications. Int. J. Biol. Macromol..

